# De NODOV-studie: een stap op weg naar gestructureerd postmortaal onderzoek bij onverwacht en onverklaard overlijden van jongvolwassenen

**DOI:** 10.1007/s12508-025-00460-6

**Published:** 2025-04-28

**Authors:** Tess M. Wemeijer, Annelotte M. Pries, Patrycja J. Puiman, Paul A. M. Hofman, Wilma L. J. M. Duijst

**Affiliations:** 1 Faculteit Recht, afdeling Strafrecht en criminologie, Maastricht University, Maastricht, Nederland; 2 Forensische Geneeskunde, GGD IJsselland, Zwolle, Nederland; 3 Algemene kindergeneeskunde, Erasmus Medisch Centrum – Sophia, Rotterdam, Nederland; 4 CAPHRI – Care and Public Health Research Institute, Faculteit Gezondheid, geneeskunde en levensstijl, Maastricht University, Maastricht, Nederland

**Keywords:** onverwacht overlijden, postmortaalonderzoeksprotocol, Wet op de lijkbezorging, Sudden unexpected death protocol, Postmortem investigation, Forensic medicine

## Abstract

Sinds 2016 is in Nederland de procedure Nader Onderzoek DoodsOorzaak bij Kinderen (NODOK) in zijn huidige vorm beschikbaar in alle Universitaire Medische Centra (UMC’s). Deze postmortaalonderzoeksprocedure kan met toestemming van ouders worden ingezet om de doodsoorzaak te achterhalen wanneer hun kind plotseling is overleden. De NODOK-procedure, waarbij in 71 % van de gevallen een doodsoorzaak werd aangetoond, heeft zijn waarde voor de nabestaanden reeds aangetoond. Mede door deze onderzoeksresultaten heeft ZonMW in 2022 een subsidie beschikbaar gesteld voor een pilot naar postmortaal onderzoek voor jongvolwassenen van 18 tot en met 45 jaar, iets wat tot op heden regionaal grote verschillen kende. In dit artikel zetten we de aanloop tot deze subsidie uiteen. Ook beschrijven we het postmortaalonderzoeksprotocol dat reeds geïmplementeerd is in Noordoost Nederland, de toekomstige verspreiding van deze pilot en de aankomende wijziging van de Wet op de Lijkbezorging.

## Abstract (en)

Since 2026, the Postmortem Evaluation of Sudden Unexplained Death in Youth (PESUDY) procedure has been available in all University Medical Centers (UMCs) in the Netherlands. This postmortem investigation in unexpected and unexplained death of minors, can be carried out with the consent of parents. The PESUDY procedure, in which a cause of death was demonstrated in 71%, has already demonstrated the valuable impact for the surviving relatives. Partly due to these PESUDY results, the Netherlands Organization of Health Research and Development (ZonMw) made funding available to implement a structured procedure for suddenly deceased young adults from 18 to 45 years old, which has shown major regional differences up to now. In this article, we explain the run-up to this funding. We also describe the postmortem research protocol that has already been implemented in the Northeast of the Netherlands, the future distribution of this pilot and the upcoming amendment of the Burial and Cremation Act.

## Inleiding

In 2022 overleden in Nederland 170.112 mensen, van wie 2.931 volwassenen tussen de twintig en 45 jaar [1]. Overleden mensen met een leeftijd tussen achttien en twintig jaar worden door het CBS niet apart gerapporteerd. Bij veel overlijdens van volwassenen tussen de twintig en 45 jaar was de oorzaak bekend, maar bij 277 jongvolwassenen was de doodsoorzaak onbekend. De betrouwbaarheid van de geregistreerde doodsoorzaken is beperkt, aangezien inwendig postmortaal onderzoek en daarmee de onderbouwing van doodsoorzaken al jaren steeds minder plaatsvindt [2].

Waar onverwachte en onverklaarde overlijdens bij jongvolwassenen vaak onbevredigend aanvoelen voor medici, roepen deze overlijdens onder minderjarigen ongerustheid op in de hoogste politieke kringen. In 2012 werd daarom in Nederland een gestructureerde postmortaalonderzoeksprocedure geïntroduceerd voor onverwachte, onverklaarde overlijdens bij minderjarigen. Deze procedure, gefinancierd door het Ministerie van Justitie en Veiligheid, stond bekend als NODO (Nader Onderzoek DoodsOorzaak). De basis van de NODO-procedure was een neutrale, niet-justitiële aanpak, bedoeld om kindermishandeling aan te tonen of uit te sluiten. Uit de resultaten van de NODO-procedure bleek dat mishandeling niet voorkwam, maar wel dat de procedure effectief was in het achterhalen van de doodsoorzaak [3]. Na evaluatie in 2016 werd de procedure aangepast en landelijk uitgerold als NODOK (Nader Onderzoek DoodsOorzaak van Kinderen) [4]. Deze procedure wordt gefinancierd door het Ministerie van Volksgezondheid, Welzijn en Sport en uitgevoerd in de zeven academische ziekenhuizen van Nederland. Bij kinderen bij wie een NODOK-procedure is uitgevoerd, werd in 71 % van de gevallen een doodsoorzaak vastgesteld [5]. Het kennen van de doodsoorzaak heeft niet alleen invloed op het rouwproces van nabestaanden, maar kan, in het geval van een erfelijke oorzaak, toekomstige vroegtijdige sterfgevallen binnen de familie helpen voorkomen [6, 7].

Geïnspireerd door het rapport *De dood als startpunt*, dat in 2017 aanbevelingen voor forensisch geneeskundig onderzoek deed, en de behaalde resultaten van de NODOK-procedure is er in 2022 incidentele financiering beschikbaar gesteld door ZonMw. Deze subsidie is bedoeld voor een pilotonderzoek ten behoeve van een gestructureerde postmortaalonderzoeksprocedure bij meerderjarigen, waarbij honderd overledenen worden onderzocht [8]. Binnen het onderzoek wordt gekeken naar de effectiviteit van de verschillende postmortale onderzoeken, welke postmortale onderzoeken de doodsoorzaken vaststellen en de efficiëntie van de gehele procedure. Dit gebeurt in aanloop naar landelijke implementatie onder de aankomende wetswijziging van de Wet op de Lijkbezorging.

## Postmortaalonderzoeksprotocol

Voor de implementatie van een gestructureerd postmortaalonderzoeksprotocol voor meerderjarigen is het bestaande NODOK-protocol voor minderjarigen als basis gebruikt. Dit protocol is aangepast en aangevuld voor toepassing op jongvolwassenen met een leeftijd van achttien tot en met 45 jaar.

Op dit moment zijn twee onderzoekscentra gerealiseerd voor de uitvoer van het onderzoeksprotocol van de NODOV-studie. De Isala Klinieken te Zwolle en het UMCG te Groningen bedienen samen zes provincies. De NODOV-procedure wordt met toestemming van de nabestaanden uitgevoerd en kan de volgende postmortale onderzoeken omvatten:

- afname van lichaamsmateriaal;
- beeldvormend onderzoek;
- obductie van het lichaam en eventueel het hoofd;
- DNA-onderzoek.

Verschillende medische specialismen dragen bij aan het vinden van de doodsoorzaak, waaronder de afdelingen algemene chemie, medische microbiologie, toxicologie, radiologie, pathologie en klinische genetica. De nabestaanden worden niet belast met kosten voor deelname aan de NODOV-studie. Ook het vervoer van en naar het onderzoekscentrum is kosteloos voor nabestaanden. De verwachte duur van de onderzoeksperiode is maximaal 24 uur, waarna het lichaam wordt teruggebracht naar de voorkeurslocatie van de nabestaanden. De uitslagen van de postmortale onderzoeken zijn na enkele weken tot maanden beschikbaar, afhankelijk van de uitgevoerde onderzoeken.

## Toekomstige verspreiding

De NODOV-studie is momenteel geïmplementeerd in Oost-Nederland (sinds 1 september 2022) en Noord-Nederland (sinds 1 januari 2024). Naar verwachting wordt het onderzoeksgebied in het tweede kwartaal van 2025 uitgebreid naar Noord-Holland, met een nieuw onderzoekscentrum in Amsterdam (zie fig. 1). Door de implementatie van de NODOV-studie bereiden de regio’s zich voor op de verwachte wijziging van de wet en de praktische organisatie van postmortale onderzoeken, op initiatief van de forensisch arts. Dit is de eerste keer dat in Nederland op een regio-overstijgende schaal onderzoek wordt gedaan naar de doodsoorzaak van onverwacht overleden jongvolwassenen. De resultaten zullen uniek zijn voor de Nederlandse situatie en bieden waarde op medisch preventief niveau, evenals voor de rouwverwerking van nabestaanden.

**Figuur 1:**
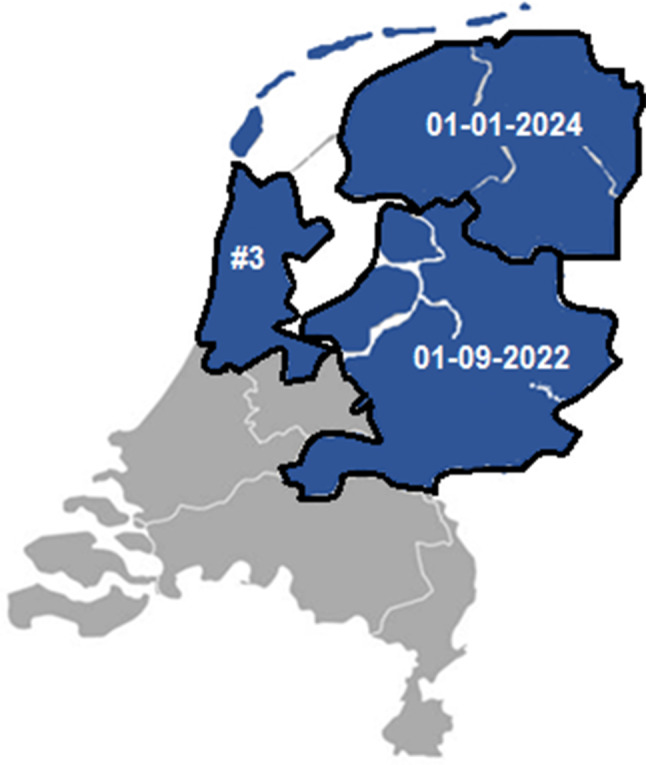
Regio’s waar de NODOV-studie is geïmplementeerd of op korte termijn wordt verwacht

## Wijziging wet op de lijkbezorging

De huidige wetgeving over overledenen en postmortale zorg, de Wet op de Lijkbezorging, stamt uit 1991 [9]. Binnen deze wet wordt sectie (obductie) als de enige mogelijkheid voor postmortaal onderzoek van een lichaam omschreven. Modernisering van de wet is noodzakelijk om meer keuzemogelijkheden te bieden voor de bestemming van het lichaam, overeenkomstig de wensen van de overledene. Daarnaast worden wijzigingen voorgesteld voor het vaststellen van de aard van het overlijden en het vaststellen van de identiteit van de overledene, met betere waarborgen voor de integriteit van het lichaam. Ook krijgt de gemeentelijke lijkschouwer meer bevoegdheden om postmortaal onderzoek in te zetten om de aard van het overlijden vast te stellen. Het conceptvoorstel voor de wetswijziging werd op 8 oktober 2024 online gepubliceerd en biedt onder andere de mogelijkheid voor radiologisch onderzoek en afname van lichaamsmateriaal voor toxicologisch onderzoek [10]. De consultatie over de voorgestelde wijzigingen is op 16 december 2024 geëindigd. Momenteel wordt het wetsvoorstel aangepast, waarna het zal worden aangeboden aan de Raad van State en de Tweede Kamer als de nieuwe wet: Wet Bestemming Lichamen en Overledenen [11].

## Conclusie

Gezien de aankomende wetswijziging is het waarschijnlijk dat de vraag naar postmortale onderzoeken aanzienlijk zal toenemen. Deze verandering zal niet alleen betrekking hebben op de forensische geneeskunde, maar ook op de pathologie, radiologie, toxicologie en zelfs huisartsen en specialisten die deze lijkschouwen doorgaans uitvoeren. De wijziging van de wet vraagt om een zorgvuldige voorbereiding om de implementatie soepel te laten verlopen. Een gestructureerde postmortaalonderzoeksprocedure is noodzakelijk om regionale verschillen in postmortaal onderzoek te verminderen en om overal dezelfde kansen en kwaliteit te waarborgen. De resultaten van de NODOV-studie leveren een waardevolle bijdrage aan de vlotte implementatie van de wet. De resultaten van deze studie zullen worden beschreven in een wetenschappelijk artikel indien de honderd beoogde overledenen zijn geïncludeerd. Naar verwachting zal dit eind 2025 het geval zijn, waarna publicatie zo spoedig mogelijk volgt.
